# Conventional MRI Markers and Cognitive Dysfunction in Multiple Sclerosis: An Exploratory Pilot Study

**DOI:** 10.1002/brb3.71868

**Published:** 2026-10-23

**Authors:** Faezeh Khorshidian, Vajiheh Aghamollaii, Maryam Noroozian, Setareh Rassa

**Affiliations:** ^1^ Cognitive Neurology, Dementia and Neuropsychiatry Research Center Tehran University of Medical Sciences Tehran Iran

**Keywords:** brain atrophy, cognitive dysfunction, magnetic resonance imaging, multiple sclerosis, neuropsychological tests

## Abstract

**Background:**

Cognitive impairment is a common and disabling manifestation of multiple sclerosis (MS), yet its structural correlates remain incompletely understood in routine clinical practice. Although advanced neuroimaging has improved our understanding of the underlying mechanisms, its limited accessibility restricts widespread clinical implementation. We aimed to investigate whether conventional MRI markers are associated with domain‐specific cognitive impairment in patients with MS.

**Methods:**

In this exploratory cross‐sectional study, 25 adults with clinically confirmed multiple sclerosis underwent cognitive assessment using the standard Persian Montreal Cognitive Assessment (MoCA), version 7.1, and conventional 1.5‐T brain MRI. Conventional MRI measures included lesion burden, lesion topography, and visually rated cortical atrophy. Associations between MRI markers and MoCA‐derived cognitive domains were assessed using Spearman rank correlation with false discovery rate adjustment for multiple testing.

**Findings:**

Twenty‐five patients with clinically confirmed MS completed cognitive and MRI assessments. Mean age was 42.4 ± 9.1 years, 56% were women, and the mean MoCA score was 22 ± 7. Periventricular (88%) and juxtacortical/cortical (84%) lesions were the most frequent MRI findings, total T2/FLAIR lesion burden showed only weak correlations with cognitive performance (ρ range −0.02 to 0.29; all FDR‐adjusted *p* > 0.65). The largest lesion‐based association was observed between infratentorial lesions and concentration (*ρ =* −0.48, unadjusted *p =* 0.018; FDR‐adjusted *p =* 0.405). Among atrophy measures, GCA showed the strongest association with visuospatial performance (*ρ =* 0.51, unadjusted *p =* 0.012; FDR‐adjusted *p =* 0.405), with additional moderate trends for concentration (*ρ =* 0.40, *p =* 0.052) and executive function (*ρ =* 0.40, *p =* 0.052). No MRI marker remained statistically significant after correction for multiple comparisons.

**Interpretation:**

Although no MRI–cognitive association remained statistically significant after correction for multiple comparisons, several unadjusted associations showed moderate effect sizes and may warrant further investigation. The strongest exploratory associations involved infratentorial lesions and concentration (*ρ =* −0.48) and global cortical atrophy and visuospatial performance (*ρ =* 0.51). The absence of consistent relationships between overall lesion burden or individual regional MRI markers and cognitive performance suggests that focal structural measures alone may not fully capture the neural substrates of cognitive dysfunction in MS. This pattern provides a rationale for further investigation of distributed, network‐level mechanisms of cognitive dysfunction, while the present findings should be considered hypothesis‐generating rather than confirmatory. Conventional MRI may therefore provide complementary structural information in the clinical assessment of cognitive dysfunction in MS. Larger, adequately powered studies using comprehensive neuropsychological assessment and quantitative, network‐sensitive MRI measures are needed to determine whether these observed patterns are reproducible and clinically meaningful.

## Graphical abstract

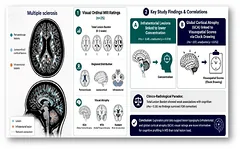

**Abbreviations**
CDT: Clock drawing test
DTI: diffusion tensor imaging
EDSS: Expanded disability status scale
fMRI: functional magnetic resonance imaging
GCA: global cortical atrophy
Gd: gadolinium
GM: grey matter
IQR: interquartile range
MRI: magnetic resonance imaging
MS: Multiple sclerosis
MTA: medial temporal atrophy
PPA: posterior parietal atrophy
PPMS: primary progressive multiple sclerosis
RRMS: relapsing–remitting multiple sclerosis
SD: standard deviation
SPMS: secondary progressive multiple sclerosis
T2/FLAIR: T2‐weighted fluid‐attenuated inversion recovery
WM: white matter

## 1 Introduction

Cognitive impairment is a major and clinically significant manifestation of multiple sclerosis, yet it remains underrecognized in routine clinical practice (Di Filippo et al. 2018; Eshaghi et al. 2018). Although MS was historically considered a primarily white matter demyelinating disorder associated with motor disability, it is now recognized as a diffuse neurodegenerative disease involving both white and grey matter, including cortical and deep grey matter pathology (Mirmosayyeb et al. 2024; Miscioscia et al. 2024; Nabizadeh et al. 2024). Neurocognitive deficits affect a substantial proportion of patients across MS phenotypes and disease stages and are major contributors to long‐term functional and socioeconomic burden (Peño et al. 2023; Jandric et al. 2022; Bonacchi et al. 2022). Cognitive impairment in MS is multidimensional, with processing speed deficits representing the most common and earliest abnormality, often accompanied by impairments in attention, working memory, executive function, and episodic memory (Petracca et al. 2021; Solana et al. 2021). Importantly, cognitive decline may occur early in the disease course, even among patients with minimal clinical disability, and is only partially captured by conventional disability measures (Cordani et al. 2021; Fujimori et al. 2020; Riccitelli et al. 2020). These findings highlight that cognitive dysfunction reflects underlying neurobiological processes not fully captured by motor‐focused clinical measures (Andravizou et al. 2019; Megna et al. 2019). At a mechanistic level, MS‐related cognitive impairment is increasingly viewed as a consequence of widespread network disruption driven by inflammatory demyelination, synaptic dysfunction, and neuroaxonal loss (Rahn et al. 2019; Pokryszko‐Dragan et al. 2018; Tóth et al. 2019). Experimental and translational studies suggest that inflammatory processes disrupt synaptic plasticity and long‐range connectivity, ultimately impairing the function of distributed cortical–subcortical networks (Eshaghi et al. 2018; Meijer et al. 2017). This network‐based framework has shifted the understanding of cognitive decline in MS beyond the traditional lesion‐centric paradigm (Meijer et al. 2017; Preziosa et al. 2017). Within this evolving framework, magnetic resonance imaging (MRI) remains central to MS diagnosis, disease monitoring, and treatment evaluation. Conventional MRI measures, including T2 lesion burden, T1 hypointense lesions, and global or regional brain atrophy, are widely available and continue to guide routine clinical decision‐making (Bisecco et al. 2018; Petracca et al. 2017; Gerstenecker et al. 2017). However, the relationship between white matter lesion burden and cognitive performance has historically been modest, reflecting the well‐recognized clinico‐radiological paradox (Mollison et al. 2017; Steenwijk et al. 2016; Preziosa et al. 2016). This paradox highlights that macroscopic lesion burden alone does not fully capture the structural basis of cognitive dysfunction (Riccitelli et al. 2020; Meijer et al. 2016).

Accumulating evidence indicates that grey matter pathology demonstrates stronger and more consistent associations with disability progression than white matter lesion load (Koenig et al. 2015; Kolind et al. 2015). Longitudinal imaging studies have shown that regional cortical thinning and subcortical volume loss follow clinically meaningful patterns. Advanced neuroimaging techniques, including diffusion tensor imaging and functional MRI connectivity analyses, have further revealed microstructural tract abnormalities and altered network organization associated with domain‐specific cognitive impairment (Yu et al. 2012; Fox et al. 2011; Filippi et al. 2011). Nevertheless, these advanced modalities remain largely limited to research settings and are not routinely used in clinical practice (Polman et al. 2011; Genova et al. 2009). Consequently, despite advances in mechanistic understanding, the clinical translation of these findings remains limited. A key unresolved question is whether widely available conventional MRI measures can meaningfully capture domain‐specific cognitive impairment when evaluated within a comprehensive framework (Benedict et al. 2007; Rovaris et al. 2008; Bisecco et al. 2015). Most prior studies have either focused on global cognitive status, single‐domain outcomes, or isolated imaging metrics (Vacchi et al. 2017; Nelson et al. 2017). Few studies have systematically examined the relative contribution of lesion burden and regional cortical atrophy across multiple cognitive domains within the same cohort using clinically accessible imaging tools. Clarifying these relationships has important clinical implications (González Torre et al. 2017; Cui et al. 2017; Spirou et al. 2018). If specific conventional MRI parameters show meaningful associations with defined cognitive domains, they may serve as pragmatic biomarkers to identify patients at risk of cognitive decline, support prognostication, and guide timely cognitive interventions (Koubiyr et al. 2019; Spiteri et al. 2019). Conversely, if cognitive dysfunction shows only weak associations with conventional imaging measures, this would further define the limitations of routine MRI in capturing clinically relevant neurodegenerative processes (Oset et al. 2020; Benedict et al. 2020).

In this study, we investigated the associations between domain‐specific cognitive impairment and conventional MRI markers, including lesion burden and regional grey matter atrophy, in patients with MS. By integrating cognitive domain assessment with clinically accessible imaging measures, we aimed to identify structural correlates of cognitive dysfunction and evaluate the potential of routine MRI as a scalable biomarker approach in MS.

## 2 Methods

### 2.1 Study Design and Setting

This exploratory cross‐sectional observational study was conducted between September 2025 and December 2025 at two tertiary academic referral centers affiliated with Tehran University of Medical Sciences, Tehran, Iran. The primary objective was to investigate the association between conventional brain MRI markers and domain‐specific cognitive impairment in patients with multiple sclerosis (MS). Given the limited sample size, the study was designed as a hypothesis‐generating exploratory analysis rather than a confirmatory investigation. The present analysis represents a secondary exploratory evaluation of data collected within a broader case‐series research protocol approved by the institutional ethics committee.

### 2.2 Participants

Adult patients with a confirmed diagnosis of MS were consecutively recruited from outpatient neurology clinics and inpatient neuropsychiatric services during the study period to minimize potential selection bias. The MS diagnosis was established according to the revised McDonald criteria by board‐certified neurologists. The study population included patients with relapsing‐remitting multiple sclerosis (RRMS) and secondary progressive multiple sclerosis (SPMS). Patients with primary progressive multiple sclerosis (PPMS) were not included in the present cohort. A total of 25 patients met the eligibility criteria and were included in the final analysis.

### 2.3 Eligibility Criteria

Participants were eligible if they:

- were ≥18 years old;
- had a definite MS diagnosis;
- had at least a high‐school education;
- completed clinical, neuropsychological, and MRI assessments.

### 2.4 Exclusion Criteria

- declined written informed consent;
- experienced clinical relapse within 30 days before evaluation;
- received high‐dose corticosteroids within 30 days before testing;
- had severe psychiatric instability;
- had neurological or medical comorbidities affecting cognition;
- were unable to complete valid cognitive testing.

### 2.5 Clinical Assessment

Collected variables included age, sex, years of education, disease duration, MS subtype, and relapse frequency. Information on disease‐modifying therapy (DMT) exposure, including current treatment status and previous treatment history, was collected when available. Neurological disability was assessed using the Expanded Disability Status Scale (EDSS) by a neurologist who was blinded to cognitive assessment results. Disability severity was categorized according to predefined EDSS ranges into mild, moderate, and severe disability groups. Disease activity was assessed based on the documented number of MS relapses. The number of attacks was recorded from clinical history and medical records.

### 2.6 Neuropsychological Assessment

Cognitive performance was assessed using the Persian validated version of the Montreal Cognitive Assessment (MoCA; version 7.1), a clinician‐administered screening instrument designed to evaluate global cognitive function across multiple domains, including attention, executive function, memory, language, visuospatial abilities, abstraction, and orientation (Rashedi et al. 2021). MoCA was selected because it provides a brief and clinically feasible assessment of cognitive function in neurological populations.

The assessment was performed under standardized clinical research conditions by trained personnel. Individual MoCA items were extracted and grouped into exploratory cognitive domains according to the neuropsychological constructs represented by the original MoCA components. The predefined item‐to‐domain mapping strategy is provided in Table 1.

For the exploratory MRI–cognition analyses, individual MoCA component‐level measures and broader cognitive domain groupings were defined a priori based on the neuropsychological constructs represented by the original MoCA components. Working memory was represented primarily by digit span performance, whereas concentration was represented by serial subtraction, and vigilance was represented by the target‐detection task. Episodic memory was assessed using delayed recall, and semantic memory using the general‐knowledge questions. Abstract reasoning was assessed using the similarity items, and visuospatial ability using the clock drawing test. Executive function was represented by the relevant executive components of the MoCA, including the alternating trail‐making task and cube copying, as specified in Table 1. These component‐level measures and broader exploratory groupings were analyzed separately as specified in the statistical analysis and tables.

The education adjustment applied in the original MoCA scoring system (+1 point for individuals with ≤12 years of education) was not applied in the present study. This decision was based on the study objective, which was not to classify participants according to the validated global MoCA cutoff but rather to investigate associations between MRI‐derived structural markers and domain‐specific cognitive performance. Therefore, analyses were performed using raw domain‐level performance measures rather than education‐adjusted total MoCA scores. Applying the education correction to individual domain‐derived variables would have introduced an artificial modification of domain‐specific scores that are not part of the original MoCA scoring framework.

Because MoCA‐derived cognitive measures and exploratory domain groupings are not equivalent to comprehensive neuropsychological test composites, they were treated as exploratory cognitive indicators. No external normative cutoff values were applied to classify participants as cognitively impaired within individual domains. Accordingly, descriptive categories reported in the tables refer to reduced performance rather than clinically diagnosed cognitive impairment and were derived from the observed performance on the corresponding MoCA component‐level measures or exploratory domain groupings. These classifications were used for descriptive purposes only, and all MRI–cognition analyses were considered hypothesis‐generating.

### 2.7 MRI Acquisition

MRI acquisition was performed using a 1.5‐T Siemens MAGNETOM Avanto scanner (Siemens Healthineers, Erlangen, Germany). The same imaging protocol was applied to all participants and included axial T2‐weighted, fluid‐attenuated inversion recovery (FLAIR), pre‐contrast T1‐weighted, and post‐contrast T1‐weighted sequences. Images were acquired with a slice thickness of 5 mm and an interslice gap of 1 mm. All examinations were performed according to the institutional standard brain MRI protocol for multiple sclerosis. For participants undergoing spinal cord evaluation, cervical and/or thoracic spinal MRI sequences were reviewed according to the available clinical imaging protocol. Spinal cord lesion presence was recorded as a binary MRI variable based on the clinical radiological report (Figure 1).

**FIGURE 1:**
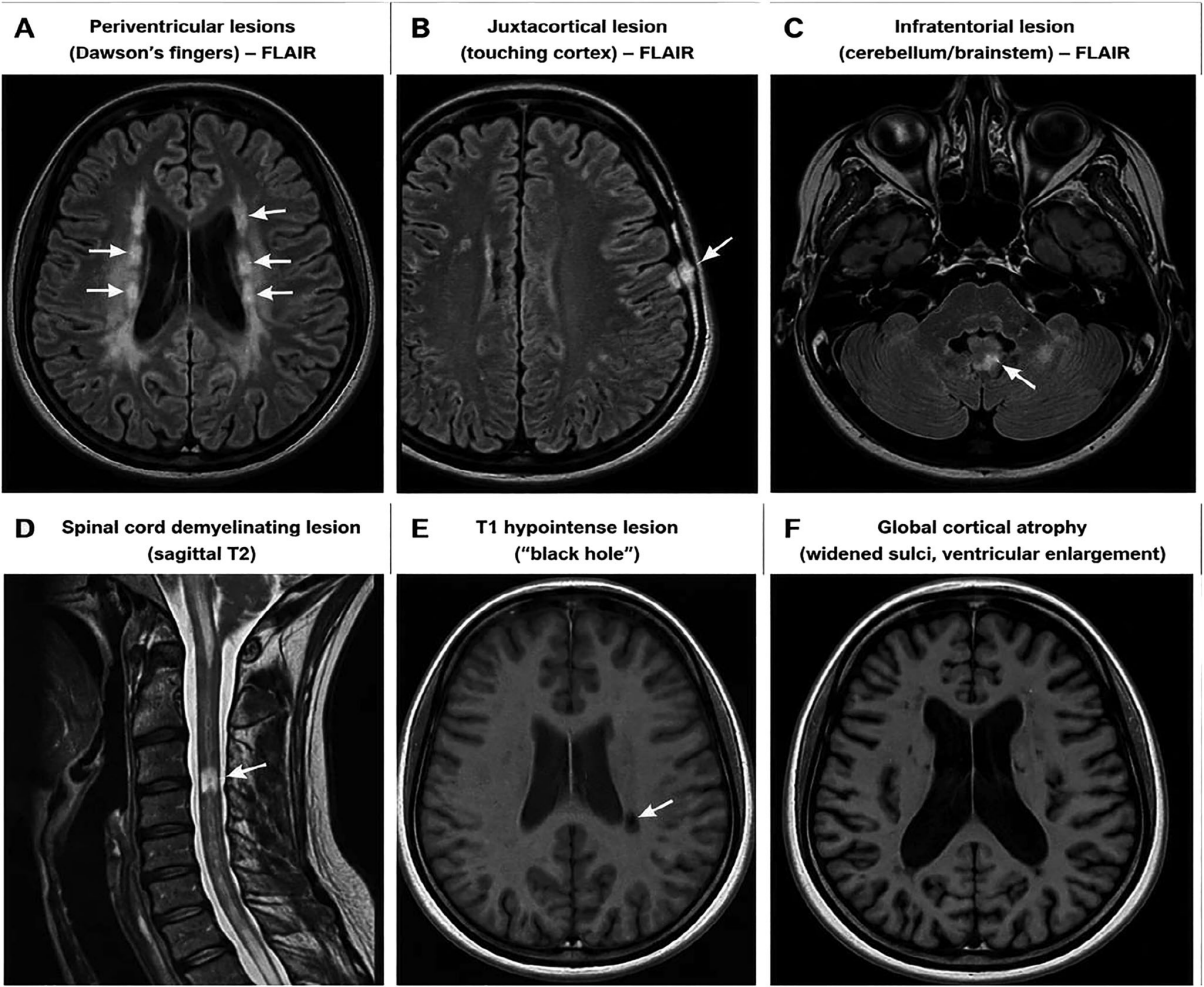
Representative conventional MRI features in multiple sclerosis (MS). This composite figure illustrates the major MRI patterns commonly observed in MS, including periventricular lesions, juxtacortical/cortical lesions, infratentorial involvement, spinal cord lesions, and regional cortical atrophy. Conventional MRI remains a practical tool for assessing disease dissemination and structural damage in MS.

### 2.8 MRI Interpretation

MRI scans were reviewed by a single experienced neuroradiologist who was blinded to all clinical and cognitive assessment data. Given that image interpretation was performed by a single reader, inter‐rater reliability could not be assessed. Intra‐rater reliability was not formally evaluated, which is acknowledged as a limitation of this exploratory study.

#### 2.8.1 Lesion Variables

The recorded MRI variables comprised total T2/FLAIR lesion burden as well as lesion distribution across periventricular, juxtacortical/cortical, infratentorial, and spinal cord regions. Lesion burden was visually rated on a 4‐point ordinal scale (0 = none, 1 = mild, 2 = moderate, 3 = severe).

#### 2.8.2 Atrophy Variables

##### 2.8.2.1 Brain Atrophy Was Assessed Using Conventional Visual Rating Scales

Global cortical atrophy (GCA) was graded according to the established Pasquier visual rating scale from 0 to 3. A score of 0 indicated normal cortical volume without ventricular enlargement; score 1 indicated mild sulcal widening and/or mild ventricular enlargement; score 2 indicated moderate cortical volume loss with moderate ventricular enlargement; and score 3 indicated severe cortical atrophy with marked volume loss and a knife‐blade appearance.

Medial temporal atrophy (MTA) was assessed on coronal T1‐weighted images through the hippocampal region. The assessment was based on three anatomical features: widening of the choroid fissure, enlargement of the temporal horn of the lateral ventricle, and reduction of hippocampal height. The MTA score ranged from 0 to 4, with higher scores indicating greater medial temporal atrophy. The highest hemispheric score was used for analysis to represent the maximum degree of medial temporal structural alteration. This approach was selected to capture the maximum degree of medial temporal structural involvement and to maintain consistency with previously described visual rating approaches.

Posterior parietal atrophy (PPA), also known as the Koedam posterior atrophy scale, was assessed visually using a grading system from 0 to 3. The most severe observed features were used for grading. Grade 0 indicated no posterior atrophy, grade 1 indicated mild sulcal widening and mild gyral atrophy, grade 2 indicated substantial sulcal widening and gyral volume loss, and grade 3 indicated marked sulcal widening and knife‐blade gyral atrophy.

### 2.9 Timing of MRI and Cognitive Assessment

MRI acquisition and cognitive assessment were performed within the same clinical evaluation period. The observed interval between MRI examination and MoCA testing ranged from 1 day to 2 months (minimum interval: 1 day; maximum interval: 2 months).

### 2.10 Outcomes

#### 2.10.1 Primary Outcome

Association between conventional MRI variables and exploratory domain‐specific cognitive performance derived from MoCA components.

#### 2.10.2 Secondary Outcomes

Associations between MRI‐derived markers and clinical severity measures, including EDSS score and relapse frequency, were assessed using Spearman rank correlation coefficients.

### 2.11 Statistical Analysis

Statistical analyses were performed using IBM SPSS Statistics version 26. Given the limited sample size, all statistical analyses were considered exploratory and hypothesis‐generating, and no causal inference was intended.

#### 2.11.1 Descriptive Analysis

- continuous variables: mean ± SD or median (IQR)
- categorical variables: frequency (%)

Data distribution was assessed using the Shapiro–Wilk test. Several variables showed significant deviations from normality; therefore, non‐parametric statistical methods were applied. Associations between MRI‐derived variables and cognitive domain measures were assessed using Spearman rank correlation coefficients (ρ).

#### 2.11.2 Primary Analysis

Associations between MRI markers and cognitive domains were assessed using Spearman rank correlation coefficients (ρ). Correlation coefficients were interpreted descriptively according to conventional effect size thresholds, with values of approximately 0.10, 0.30, and 0.50 representing small, moderate, and large effects, respectively Cohen (1988).

#### 2.11.3 Secondary Analyses

Group comparisons were performed using the Mann–Whitney U test, Kruskal–Wallis test, and χ^2^ test or Fisher's exact test, as appropriate.

#### 2.11.4 Multiple Comparisons

Because multiple MRI variables and cognitive domains were evaluated, correction for multiple comparisons was performed using both the Benjamini–Hochberg false discovery rate (FDR) method and the Holm–Bonferroni procedure. FDR‐adjusted p‐values are presented in the main tables, whereas complete Holm–Bonferroni adjusted results are provided in Table 2. Statistical significance was defined as an adjusted p‐value <0.05.

### 2.12 Ethical Approval

The study followed the principles of the Declaration of Helsinki. Written informed consent was obtained from all participants. The study protocol, originally approved as a case‐series project, was reviewed and approved by the Ethics Committee of Tehran University of Medical Sciences (Approval code: IR.TUMS.MEDICINE.REC.1405.133). The present work represents a secondary analysis of the approved dataset.

## 3 Results

Twenty‐five participants with complete MRI and cognitive assessment data were included in the correlation analyses. The demographic, clinical, cognitive, and MRI characteristics of the study population are summarized in Table 1. Spearman correlation analyses were performed to evaluate the associations between MRI‐derived structural markers, cognitive domain scores, disability severity (EDSS), and disease activity measured by the number of MS relapses.

**TABLE 1:** Baseline demographic, clinical, cognitive, and MRI characteristics of the study population (n = 25).

Characteristic	Study population (*n* = 25)
Age, years, mean ± SD / median (IQR)	42.4 ± 9.1
Female sex, n (%)	14 (56)
Education level, n (%)	
High school	2 (8)
diploma	15 (60)
University education	8 (32)
Disease duration, years	
< 1 y	1 (4)
1–5 y	7 (28)
6‐10 y	9 (36)
>10 y	8 (32)
MS phenotype, n (%)	
RRMS	14 (56)
SPMS	11 (44)
PPMS	0 (0)
EDSS severity category, n (%)	
Mild	13 (52)
Moderate	9 (36)
Severe	3 (12)
Number of relapses, n (%)	
0–1	8 (32)
2–3	13 (52)
≥4	4 (16)
Disease‐modifying therapy, n (%)	
Interferon beta‐1a	4 (16)
Ocrelizumab	8 (32)
Rituximab	6 (24)
Fingolimod	2 (8)
Dimethyl fumarate	3 (12)
Teriflunomide	2 (8)
Total MoCA score, mean ± SD	22 ± 7
Cognitive impairment domains, n (%)	
Attention/concentration impairment	14 (56)
Memory impairment	13 (52)
Executive dysfunction	13 (52)
Language impairment	3 (12)
Reduced performance across multiple cognitive domains/components	15 (60)
MRI lesion burden category, n (%)	
Periventricular lesions	22 (88)
Juxtacortical/cortical lesions	21 (84)
Infratentorial lesions	11 (44)
Spinal cord lesions	10 (40)
gadolinium‐enhancing lesions	3 (12)
Atrophy findings, n (%)	
Global cortical atrophy	14 (56)
Medial temporal atrophy	9 (36)
Posterior parietal atrophy	6 (24)

Baseline demographic, clinical, cognitive, and MRI characteristics of the study population are summarized in Table 1. The mean age of participants was 42.4 ± 9.1 years, and 56% were female. Most participants had relapsing‐remitting multiple sclerosis (56%), while 44% had secondary progressive multiple sclerosis. No participants with primary progressive multiple sclerosis were included. EDSS severity categories included mild disability in 13 participants (52%), moderate disability in 9 participants (36%), and severe disability in 3 participants (12%). Cognitive assessment showed a mean total MoCA score of 22 ± 7. Exploratory MoCA‐based domain analysis identified reduced performance across multiple cognitive domains in 60% of participants. Conventional MRI evaluation demonstrated frequent involvement of periventricular and juxtacortical/cortical regions, while gadolinium‐enhancing lesions were observed in a minority of participants.

Associations between MRI‐derived structural markers and exploratory MoCA‐based cognitive domain scores are presented in Table 2. The complete Holm–Bonferroni adjusted p‐values are provided in Table 2. Spearman correlation analyses were performed, and p‐values were adjusted for multiple comparisons using both the Benjamini–Hochberg false discovery rate (FDR) and Holm–Bonferroni procedures.

**TABLE 2:** Association between MRI indices and cognitive domains after multiple‐comparison correction.

2.1 MRI T2/FLAIR lesion load			
Cognitive domain	Spearman ρ	Unadjusted p‐value	FDR‐adjusted p
**Concentration**	0.04	0.849	0.926
**Working memory**	0.29	0.172	0.653
**Vigilance**	0.29	0.172	0.653
**Episodic memory**	0.25	0.232	0.653
**Semantic memory**	0.10	0.643	0.844
**Executive function**	−0.02	0.926	0.947
**Visuospatial**	0.11	0.610	0.844
**Abstract reasoning**	0.12	0.575	0.844
**Multidomain impairment**	0.16	0.455	0.756

2.2 Juxtacortical/cortical lesions			
Cognitive domain	Spearman ρ	Unadjusted p‐value	FDR‐adjusted p
**Concentration**	0.35	0.097	0.653
**Working memory**	0.12	0.591	0.844
**Vigilance**	0.20	0.349	0.706
**Episodic memory**	0.08	0.713	0.870
**Semantic memory**	0.09	0.665	0.844
**Executive function**	−0.04	0.862	0.926
**Visuospatial**	0.29	0.174	0.653
**Abstract reasoning**	0.17	0.430	0.756
**Multidomain impairment**	0.35	0.097	0.653

2.3 Periventricular lesions			
Cognitive domain	Spearman ρ	Unadjusted p‐value	FDR‐adjusted p
**Concentration**	0.29	0.165	0.653
**Working memory**	0.03	0.880	0.926
**Vigilance**	0.17	0.430	0.756
**Episodic memory**	0.00	1.000	1.000
**Semantic memory**	0.08	0.714	0.870
**Executive function**	0.09	0.659	0.844
**Visuospatial**	0.24	0.253	0.653
**Abstract reasoning**	0.14	0.505	0.791
**Multidomain impairment**	0.23	0.246	0.653

2.4 Infratentorial lesions			
Cognitive domain	Spearman ρ	Unadjusted p‐value	FDR‐adjusted p
**Concentration**	−0.48	0.018	0.405
**Working memory**	0.04	0.840	0.926
**Vigilance**	0.08	0.726	0.870
**Episodic memory**	−0.06	0.781	0.900
**Semantic memory**	−0.18	0.410	0.756
**Executive function**	−0.10	0.646	0.844
**Visuospatial**	−0.17	0.426	0.756
**Abstract reasoning**	−0.32	0.128	0.653
**Multidomain impairment**	0.14	0.367	0.706

2.5 Global cortical atrophy			
Cognitive domain	Spearman ρ	Unadjusted p‐value	FDR‐adjusted p
**Concentration**	0.40	0.052	0.585
**Working memory**	0.08	0.719	0.870
**Vigilance**	0.26	0.249	0.653
**Episodic memory**	0.34	0.104	0.653
**Semantic memory**	0.27	0.224	0.653
**Executive function**	0.40	0.052	0.585
**Visuospatial**	0.51	0.012	0.405
**Abstract reasoning**	0.21	0.334	0.706
**Multidomain impairment**	0.12	0.640	0.844

Overall, none of the MRI–cognitive associations remained statistically significant after correction for multiple comparisons. T2/FLAIR lesion load showed weak correlations with individual cognitive domains, with the largest correlations observed for working memory and vigilance (both *ρ =* 0.29, unadjusted *p =* 0.172), although these findings were not significant after adjustment. These analyses were considered exploratory given the limited sample size and the number of cognitive domains assessed.

Juxtacortical/cortical lesion burden demonstrated weak‐to‐moderate correlations with concentration and multidomain impairment (both *ρ =* 0.35, unadjusted *p =* 0.097), but no association remained significant after FDR or Holm correction. Periventricular lesion burden showed no significant associations with cognitive domains.

Infratentorial lesion burden demonstrated a moderate negative correlation with concentration (*ρ =* −0.48, unadjusted *p =* 0.018); however, this association was not significant after FDR correction (adjusted *p =* 0.405) or Holm correction (adjusted *p =* 0.792). Global cortical atrophy showed a moderate positive correlation with visuospatial scores measured by CDT (*ρ =* 0.51, unadjusted *p =* 0.012); however, this association did not remain significant after multiple‐comparison correction. Other evaluated atrophy measures, including MTA and PPA, showed no significant corrected associations.

Associations between MRI‐derived markers and clinical severity measures, including EDSS score and number of MS attacks, are presented in Table 3. The complete Holm–Bonferroni adjusted results for MRI–clinical severity associations are provided in Table 2. Spearman correlation analyses were performed with adjustment for multiple comparisons using FDR and Holm–Bonferroni procedures.

**TABLE 3:** Association between EDSS, MS attacks, and MRI indices after multiple‐comparison correction.

3.1. Lesion‐based MRI markers				
MRI marker	Severity variable	Spearman ρ	Unadjusted p‐value	FDR‐adjusted p
**T2/FLAIR lesion load**	EDSS score	0.011	0.959	0.959
**T2/FLAIR lesion load**	Number of MS attacks	0.164	0.445	0.959
**Juxtacortical/cortical lesions**	EDSS score	0.017	0.938	0.959
**Juxtacortical/cortical lesions**	Number of MS attacks	0.482	0.017	0.306
**Periventricular lesions**	EDSS score	0.123	0.568	0.959
**Periventricular lesions**	Number of MS attacks	0.374	0.072	0.648
**Infratentorial lesions**	EDSS score	−0.139	0.517	0.959
**Infratentorial lesions**	Number of MS attacks	−0.207	0.331	0.959
**Spinal cord lesions**	EDSS score	−0.090	0.675	0.959
**Spinal cord lesions**	Number of MS attacks	0.013	0.953	0.959

3.2 Gadolinium‐enhancing lesions				
MRI marker	Severity variable	Spearman ρ	Unadjusted p‐value	FDR‐adjusted p
**gadolinium‐enhancing lesions**	EDSS score	0.023	0.917	0.959
**gadolinium‐enhancing lesions**	Number of MS attacks	−0.090	0.677	0.959

3.3 Atrophy‐based MRI markers				
MRI marker	Severity variable	Spearman ρ	Unadjusted p‐value	FDR‐adjusted p
**Global cortical atrophy, GCA score**	EDSS score	0.117	0.587	0.959
**Global cortical atrophy, GCA score**	Number of MS attacks	0.045	0.834	0.959
**Medial temporal atrophy, MTA score**	EDSS score	0.162	0.451	0.959
**Medial temporal atrophy, MTA score**	Number of MS attacks	−0.228	0.285	0.959
**Posterior parietal atrophy, PPA score**	EDSS score	−0.035	0.872	0.959
**Posterior parietal atrophy, PPA score**	Number of MS attacks	−0.283	0.181	0.959

After correction, none of the MRI–clinical severity associations remained statistically significant. T2/FLAIR lesion load showed no significant correlation with EDSS score or number of MS attacks. Juxtacortical/cortical lesions showed a moderate positive correlation with the number of MS attacks (*ρ =* 0.482, unadjusted *p =* 0.017); however, this association did not remain significant after FDR or Holm correction.

Periventricular lesions showed a moderate but non‐significant correlation with relapse frequency (*ρ =* 0.374, unadjusted *p =* 0.072), whereas infratentorial and spinal cord lesion measures showed no significant associations with EDSS score or number of MS attacks. Similarly, gadolinium‐enhancing lesions were not significantly associated with either clinical severity measure.

None of the atrophy‐related markers, including GCA, MTA, and PPA, demonstrated significant associations with EDSS score or relapse frequency after multiple‐comparison correction.

## 4 Discussion

Our exploratory cross‐sectional study examined whether routinely available conventional MRI markers—including overall lesion burden, lesion topography, and visually rated cortical atrophy—were associated with domain‐specific cognitive performance in patients with multiple sclerosis (MS). After adjustment for multiple comparisons, none of the observed MRI–cognitive associations remained statistically significant. Nevertheless, several associations demonstrated moderate effect sizes that may represent biologically meaningful patterns deserving further investigation in adequately powered studies. Rather than providing confirmatory evidence, our findings contribute to the growing body of literature suggesting that the relationship between conventional MRI abnormalities and cognitive dysfunction in MS is complex and extends beyond overall lesion accumulation.

A notable finding was the absence of meaningful associations between total T2/FLAIR lesion burden and most cognitive domains. Although modest correlations were observed for selected domains before adjustment, these did not persist after correction for multiple testing. This observation is consistent with the well‐established clinico‐radiological paradox in MS, whereby the extent of white matter lesions visible on conventional MRI explains only a limited proportion of inter‐individual variability in cognitive performance (Oset et al. 2020; Benedict et al. 2020). In contrast to overall lesion burden, lesion topography appeared to show relatively stronger exploratory associations with cognition. Among lesion‐based variables, infratentorial lesions demonstrated the largest effect size, showing a moderate inverse association with attention/concentration before multiple‐comparison correction. Although this association was no longer statistically significant after adjustment, its magnitude is consistent with previous work highlighting the contribution of cerebellar and brainstem pathways to attentional control, executive coordination, and cognitive timing (Oset et al. 2020; Meng et al. 2021; Rocca et al. 2022).

Experimental and connectomic studies have shown that lesions affecting posterior fossa structures may disrupt widespread cortico‐cerebellar and brainstem‐cortical networks, thereby influencing cognitive performance beyond the local lesion itself (Benedict et al. 2020; Rocca et al. 2022). Our findings should therefore be interpreted as exploratory support for the hypothesis that lesion location may be more informative than total lesion burden when investigating cognitive dysfunction in MS. Among the atrophy measures evaluated, global cortical atrophy (GCA) demonstrated the strongest association with cognitive performance, particularly for visuospatial function, although this relationship also did not remain significant after correction for multiple comparisons. Because higher visuospatial scores represented better performance in our scoring system, the observed positive correlation likely reflects the exploratory nature of the domain derivation and the limited sample rather than a straightforward biological relationship. Consequently, this result should be interpreted cautiously and should not be viewed as evidence that greater cortical atrophy is associated with better cognitive performance. Nonetheless, the observation that GCA showed one of the largest effect sizes among the evaluated MRI markers is broadly consistent with previous studies suggesting that diffuse cortical neurodegeneration may be more closely related to cognitive dysfunction than conventional white matter lesion burden (Benedict et al. 2020; Schoonheim et al. 2022). Future studies using quantitative volumetric measures and larger cohorts will be necessary to clarify the direction and strength of these relationships.

Regional visual atrophy measures, including medial temporal atrophy (MTA) and posterior parietal atrophy (PPA), were not associated with domain‐specific cognitive performance in the present cohort. Several explanations may account for these findings. First, MS‐related cognitive dysfunction is increasingly considered a network‐level disorder involving simultaneous alterations in white matter connectivity, cortical integrity, and deep grey matter structures rather than isolated regional degeneration (Meng et al. 2021; Rocca et al. 2022; Schoonheim et al. 2022; Prouskas et al. 2022). Second, visual rating scales, while practical for routine clinical use, are inherently less sensitive than quantitative volumetric techniques for detecting subtle regional neurodegeneration. Finally, the relatively small and clinically heterogeneous sample may have limited the ability to detect weaker regional associations. Similarly, we did not observe statistically significant relationships between conventional MRI markers and clinical severity measures, including EDSS score and relapse frequency, after correction for multiple comparisons. Moderate unadjusted correlations involving juxtacortical lesions and relapse frequency should therefore be regarded as hypothesis‐generating observations rather than confirmed associations. These findings are consistent with previous reports showing that disability progression and relapse activity are influenced by multiple pathological processes that are incompletely captured by conventional structural MRI alone (Langley et al. 2023; Carter et al. 2023).

Taken together, our findings reinforce the notion that conventional MRI provides only a partial representation of the mechanisms underlying cognitive dysfunction in MS. While routine imaging remains indispensable for diagnosis and disease monitoring, conventional structural measures appear insufficient to fully explain cognitive variability across individuals (Galus et al. 2026; Patitucci et al. 2023; Marselli et al. 2024). Advanced MRI techniques, including volumetric analyses, diffusion‐based imaging, and functional connectivity assessment, have consistently demonstrated stronger relationships with cognitive performance because they better characterize diffuse neuroaxonal injury and network disruption (Prouskas et al. 2022; Oreja‐Guevara et al. 2019). Nevertheless, these techniques are not yet universally available in routine clinical practice. From this perspective, our study suggests that conventional MRI markers—particularly lesion topography and visually assessed cortical atrophy—may still provide complementary structural information when interpreted alongside standardized cognitive assessment, although they should not be considered reliable surrogate markers of cognitive impairment on their own (Matias‐Guiu et al. 2018).

Several strengths should also be acknowledged. We evaluated multiple conventional MRI characteristics within the same cohort while examining several cognitive domains using a standardized cognitive screening instrument. In addition, correction for multiple comparisons reduced the likelihood of false‐positive findings and allowed a more conservative interpretation of the observed associations. Although this approach resulted in the loss of statistical significance for individual associations, reporting both effect sizes and adjusted analyses provides a more transparent assessment of the exploratory nature of the findings.

Overall, our results neither support a strong relationship between conventional MRI abnormalities and domain‐specific cognition nor suggest that routine MRI is sufficient for identifying cognitive dysfunction in individual patients. Instead, they indicate that certain structural patterns—particularly lesion distribution and global cortical atrophy—may warrant further investigation as potential components of multimodal biomarker models. Confirmation of these preliminary observations will require larger longitudinal studies using comprehensive neuropsychological assessment together with quantitative neuroimaging techniques.

## 5 Limitations

Several limitations should be considered when interpreting the findings of this exploratory study.

First, the relatively small sample size limited statistical power and resulted in wide uncertainty around effect estimates. Therefore, the observed associations should be interpreted as hypothesis‐generating findings rather than definitive evidence. The limited sample size also restricted the ability to perform multivariable analyses or adequately account for potential confounding factors.

Second, the cross‐sectional design prevents evaluation of temporal relationships between MRI abnormalities and cognitive trajectories. Longitudinal studies are required to determine whether conventional MRI markers can predict future cognitive decline or progression.

Third, MRI assessment was based on conventional 1.5‐T imaging and visual rating scales rather than quantitative volumetric analysis, cortical thickness measurements, diffusion imaging, or functional connectivity approaches. Therefore, subtle microstructural abnormalities and network‐level alterations contributing to cognitive dysfunction may not have been detected.

Fourth, although information regarding disease‐modifying therapy exposure was collected, the limited sample size and heterogeneity of treatment regimens prevented reliable assessment of treatment‐related effects on MRI findings and cognition.

Additional limitations include single‐reader MRI evaluation without formal reliability assessment, the use of exploratory MoCA‐derived cognitive domains rather than comprehensive neuropsychological composite scores, and incomplete adjustment for cognitive reserve‐related factors such as occupational complexity and lifestyle characteristics.

Finally, although appropriate multiple‐comparison correction methods were applied, the exploratory design involving multiple MRI markers and cognitive domains requires cautious interpretation. Future larger multicenter studies are needed to validate these preliminary observations.

## 6 Future Directions

Future studies should investigate the structural and network mechanisms underlying cognitive dysfunction in multiple sclerosis through larger, multicenter longitudinal designs.

Integration of conventional MRI with quantitative measures of brain volume, cortical thickness, diffusion‐based imaging, and functional connectivity analysis may provide a more comprehensive characterization of the relationship between focal lesions, diffuse neurodegeneration, and cognitive decline.

Further research should particularly examine whether lesion topology, including infratentorial involvement, and patterns of cortical atrophy provide incremental value beyond conventional lesion burden measures. Network‐based approaches focusing on cerebellar, thalamocortical, and frontoparietal systems may help explain individual differences in cognitive outcomes despite similar visible lesion loads.

Future investigations should also incorporate cognitive reserve measures, including education, occupational complexity, and lifestyle factors, to better understand variability in cognitive resilience among patients with comparable structural disease burden.

Ultimately, combining accessible MRI markers with standardized cognitive assessment and advanced imaging biomarkers may contribute to clinically practical multimodal models for early identification and monitoring of cognitive dysfunction in multiple sclerosis.

## 7 Conclusion

Conventional MRI markers showed several exploratory associations with domain‐specific cognitive performance in MS, with regional lesion distribution—particularly infratentorial involvement—and global cortical atrophy showing larger observed effect sizes than total lesion burden alone. However, none of the MRI–cognitive associations remained statistically significant after correction for multiple comparisons. The observed pattern suggests that regional lesion distribution and cortical atrophy may warrant further investigation as complementary structural correlates of cognitive performance beyond overall lesion burden. These findings should be considered hypothesis‐generating rather than confirmatory. Conventional MRI may provide complementary structural information when interpreted together with cognitive assessment; however, validation in larger, adequately powered longitudinal cohorts using comprehensive neuropsychological evaluation and quantitative imaging approaches is required.

## 8 Key Notes

*Cognitive dysfunction is a frequent and clinically relevant manifestation of multiple sclerosis, but its structural correlates are not fully captured by routine MRI lesion burden measures.
*This exploratory study investigated the relationship between conventional MRI markers, including lesion burden, lesion distribution, and visually assessed brain atrophy, and domain‐specific cognitive performance in patients with multiple sclerosis.
*Total T2/FLAIR lesion burden showed limited associations with cognitive performance, while several regional and atrophy‐related measures showed moderate unadjusted effect sizes.
*The strongest exploratory lesion‐based association involved infratentorial lesions and concentration performance, suggesting that regional lesion distribution may warrant further investigation as a potential correlate of cognitive performance beyond overall lesion burden.
*Global cortical atrophy showed a moderate unadjusted association with visuospatial performance; however, this association did not remain statistically significant after correction for multiple comparisons and should be considered an exploratory observation requiring confirmation.
*None of the MRI–cognitive associations remained statistically significant after FDR or Holm–Bonferroni correction for multiple comparisons. The observed effect‐size pattern is therefore hypothesis‐generating rather than confirmatory.
*The observed pattern is consistent with the possibility that cognitive dysfunction in multiple sclerosis may involve distributed structural changes, including regional lesion distribution and cortical atrophy; however, this hypothesis requires confirmation in larger studies.
*Conventional MRI may provide complementary information when integrated with cognitive assessment but should not be considered a standalone biomarker of cognitive dysfunction.
*Larger, adequately powered longitudinal studies combining comprehensive neuropsychological assessment with quantitative and network‐sensitive MRI techniques are needed to determine whether these exploratory patterns are reproducible and clinically meaningful.

## Author Contributions

**Faezeh Khorshidian**: study concept and design, acquisition of data, interpretation of data, writing – original draft. **Vajiheh Aghamollaii**: acquisition of data, writing – review and editing. **Maryam Noroozian**: acquisition of data, writing – review and editing. **Setareh Rassa**: acquisition of data.

## Funding

The authors have nothing to report.

## Ethics Statement

The study protocol, originally approved as a case‐series project, was reviewed and approved by the Ethics Committee of Tehran University of Medical Sciences (Approval code: IR.TUMS.MEDICINE.REC.1405.133). The present work represents a secondary analysis of the approved dataset.

## Conflicts of Interest

The authors declare that there is no conflicts of interest.

## Supporting information




**Supplementary Table 1**: Mapping of standard Persian MoCA components to the exploratory cognitive measures used in the present study
**Supplementary Table 2**: Holm–Bonferroni adjusted p‐values for MRI–cognitive and MRI–clinical severity associations

## Data Availability

The complete data of this study is available upon request from the authors.
