# Supplementary material for: Conventional MRI Markers and Cognitive Dysfunction in Multiple Sclerosis: An Exploratory Pilot Study
Source: Brain Behav. 2026 Oct 23;16(10):e71868. doi: 10.1002/brb3.71868 (PMC13691536; doi:10.1002/brb3.71868)
Supplement: Supplementary file 1 [file BRB3-16-e71868-s001.docx]

**Supplementary Table 1. Mapping of standard Persian MoCA components to the exploratory cognitive measures used in the present study**

| Exploratory cognitive measure | Standard Persian MoCA component(s) used | Possible score range | Direction of scoring |
| --- | --- | --- | --- |
| Working memory | Digit span (forward and backward) | 0–2 | Higher = better performance |
| Concentration | Serial 7 subtraction | 0–3 | Higher = better performance |
| Vigilance | Target-detection task (tap response to the specified target stimulus) | 0–1 | Higher = better performance |
| Episodic memory | Delayed recall | 0–5 | Higher = better performance |
| Semantic memory | Two general-knowledge questions | 0–2 | Higher = better performance |
| Executive function | Alternating trail-making task and cube copying | 0-2 | Higher = better performance |
| Visuospatial ability | Clock Drawing Test | 0-3 | Higher = better performance |
| Abstract reasoning | Similarity items | 0–2 | Higher = better performance |
| *Reduced performance across multiple cognitive domains/components | Reduced performance across more than one exploratory cognitive domain/component | Not applicable | — |

Note: All cognitive measures were derived from the standard Persian Montreal Cognitive Assessment (MoCA), version 7.1, administered according to the standard instrument. The component-level measures and broader domain groupings presented here were defined a priori for the exploratory MRI–cognition analyses and do not represent independently validated neuropsychological composites. Higher scores indicate better cognitive performance. No external normative cutoff values were applied to the exploratory component-level measures or domain groupings.

*Operational definition of reduced performance: Reduced performance for an individual MoCA-derived cognitive measure was defined as a score below the maximum possible score for that measure. Reduced performance across multiple cognitive domains/components was defined as reduced performance in two or more of the prespecified exploratory cognitive measures/domains. These classifications were used for descriptive and exploratory analyses only and were not intended to indicate clinically diagnosed cognitive impairment.

**Supplementary Table 2. Holm–Bonferroni adjusted p-values for MRI–cognitive and MRI–clinical severity associations**

**A. MRI–cognitive domain associations (n = 25)**

| MRI marker | Cognitive domain | Holm–Bonferroni adjusted p-value |
| --- | --- | --- |
| T2/FLAIR lesion load | Concentration | 1.000 |
| T2/FLAIR lesion load | Working memory | 1.000 |
| T2/FLAIR lesion load | Vigilance | 1.000 |
| T2/FLAIR lesion load | Episodic memory | 1.000 |
| T2/FLAIR lesion load | Semantic memory | 1.000 |
| T2/FLAIR lesion load | Executive function | 1.000 |
| T2/FLAIR lesion load | Visuospatial ability | 1.000 |
| T2/FLAIR lesion load | Abstract reasoning | 1.000 |
| T2/FLAIR lesion load | Reduced performance across multiple cognitive domains/components | 1.000 |
| Juxtacortical/cortical lesions | Concentration | 1.000 |
| Juxtacortical/cortical lesions | Working memory | 1.000 |
| Juxtacortical/cortical lesions | Vigilance | 1.000 |
| Juxtacortical/cortical lesions | Episodic memory | 1.000 |
| Juxtacortical/cortical lesions | Semantic memory | 1.000 |
| Juxtacortical/cortical lesions | Executive function | 1.000 |
| Juxtacortical/cortical lesions | Visuospatial ability | 1.000 |
| Juxtacortical/cortical lesions | Abstract reasoning | 1.000 |
| Juxtacortical/cortical lesions | Reduced performance across multiple cognitive domains/components | 1.000 |
| Periventricular lesions | Concentration | 1.000 |
| Periventricular lesions | Working memory | 1.000 |
| Periventricular lesions | Vigilance | 1.000 |
| Periventricular lesions | Episodic memory | 1.000 |
| Periventricular lesions | Semantic memory | 1.000 |
| Periventricular lesions | Executive function | 1.000 |
| Periventricular lesions | Visuospatial ability | 1.000 |
| Periventricular lesions | Abstract reasoning | 1.000 |
| Periventricular lesions | Reduced performance across multiple cognitive domains/components | 1.000 |
| Infratentorial lesions | Concentration | 0.792 |
| Infratentorial lesions | Working memory | 1.000 |
| Infratentorial lesions | Vigilance | 1.000 |
| Infratentorial lesions | Episodic memory | 1.000 |
| Infratentorial lesions | Semantic memory | 1.000 |
| Infratentorial lesions | Executive function | 1.000 |
| Infratentorial lesions | Visuospatial ability | 1.000 |
| Infratentorial lesions | Abstract reasoning | 1.000 |
| Infratentorial lesions | Reduced performance across multiple cognitive domains/components | 1.000 |
| Global cortical atrophy | Concentration | 1.000 |
| Global cortical atrophy | Working memory | 1.000 |
| Global cortical atrophy | Vigilance | 1.000 |
| Global cortical atrophy | Episodic memory | 1.000 |
| Global cortical atrophy | Semantic memory | 1.000 |
| Global cortical atrophy | Executive function | 1.000 |
| Global cortical atrophy | Visuospatial ability | 0.540 |
| Global cortical atrophy | Abstract reasoning | 1.000 |
| Global cortical atrophy | Reduced performance across multiple cognitive domains/components | 1.000 |

**B. MRI–clinical severity associations (n = 25)**

| MRI marker | Clinical variable | Holm–Bonferroni adjusted p-value |
| --- | --- | --- |
| T2/FLAIR lesion load | EDSS score | 1.000 |
| T2/FLAIR lesion load | Number of MS attacks | 1.000 |
| Juxtacortical/cortical lesions | EDSS score | 1.000 |
| Juxtacortical/cortical lesions | Number of MS attacks | 0.306 |
| Periventricular lesions | EDSS score | 1.000 |
| Periventricular lesions | Number of MS attacks | 1.000 |
| Infratentorial lesions | EDSS score | 1.000 |
| Infratentorial lesions | Number of MS attacks | 1.000 |
| Spinal cord lesions | EDSS score | 1.000 |
| Spinal cord lesions | Number of MS attacks | 1.000 |
| Gadolinium-enhancing lesions | EDSS score | 1.000 |
| Gadolinium-enhancing lesions | Number of MS attacks | 1.000 |
| Global cortical atrophy (GCA score) | EDSS score | 1.000 |
| Global cortical atrophy (GCA score) | Number of MS attacks | 1.000 |
| Medial temporal atrophy (MTA score) | EDSS score | 1.000 |
| Medial temporal atrophy (MTA score) | Number of MS attacks | 1.000 |
| Posterior parietal atrophy (PPA score) | EDSS score | 1.000 |
| Posterior parietal atrophy (PPA score) | Number of MS attacks | 1.000 |

Holm–Bonferroni adjusted p-values were calculated for all MRI–cognitive and MRI–clinical severity associations. Due to extensive multiple-comparison correction and the exploratory nature of the analysis, most adjusted values reached 1.000. These results are provided as supplementary material, while FDR-adjusted p-values are presented in the main tables for readability.
